# The Role of BMI, Body Fat Mass and Visceral Fat in Executive Function in Individuals with Overweight and Obesity

**DOI:** 10.3390/nu13072259

**Published:** 2021-06-30

**Authors:** Miriam Sánchez-SanSegundo, Ana Zaragoza-Martí, Iciar Martin-LLaguno, Marina Berbegal, Rosario Ferrer-Cascales, José Antonio Hurtado-Sánchez

**Affiliations:** 1 Department of Health Psychology, Faculty of Health Science, University of Alicante, 03690 Alicante, Spain; miriam.sanchez@ua.es (M.S.-S.); marinaberbe@hotmail.com (M.B.); rosario.ferrer@ua.es (R.F.-C.); 2 Department of Nursing, Faculty of Health Science University of Alicante, 03690 Alicante, Spain; ja.hurtado@ua.es; 3 Alicante Institute for Health and Biomedical Research (ISABIAL-FISABIO Foundation), 03010 Alicante, Spain; 4 Faculty of Health Science, University of Alicante, 03690 Alicante, Spain; iciar.martin@ua.es

**Keywords:** overweight, obesity, executive function, adiposity

## Abstract

Evidence accumulated to date suggests that excess weight in the adult population is associated with a wide range of impairments in executive function. However, most studies have only examined the influence of body mass index (BMI) on the cognitive function of individuals with overweight and obesity. This study examined the potential associations of markers of adiposity (BMI, body fat, and visceral fat) with five domains of executive function including cognitive flexibility, inhibition, monitoring, planning, and working memory in a sample of 87 adult with overweight (*n* = 34) and obesity (*n* = 53). The results show that obese people had poorer working memory than those with overweight. After controlling for educational levels and physical activity, the results suggest that neither the waist–hip index not visceral fat were associated with cognitive function. In overweight, body fat was negatively associated with executive components of inhibition (*p* = 0.05) and monitoring (*p* = 0.02). In the obesity subgroup, body fat was negatively associated with inhibition (0.02) and working memory (0.04). The results provide evidence of the importance of adiposity for cognitive function. The implications for understanding the influence of markers of adiposity in adults with overweight and obesity are discussed.

## 1. Introduction

Overweight and obesity are significant global public health problems. Their prevalence has increased dramatically over the past three decades in many regions of the world (WHO 2007). Epidemiological studies have demonstrated that a high body mass index (BMI) is associated with an increased risk of premature mortality and disability, accounting for about 4 million deaths and 120 million cases of disability per year globally [1].

Overweight and obesity have been associated with the development of numerous diseases, including cardiovascular diseases [2], diabetes mellitus [3], stroke [4] (Winter et al., 2008), and some cancers [5]. Recent evidence has also demonstrated that excess weight in the adult population predicts cognitive decline and is one of the main risk factors for the development of neurodegenerative disorders, particularly vascular dementia and Alzheimer’s disease [6]. Although the biological mechanisms implicated in the pathogenesis of cognitive impairment are not fully understood, the evidence accumulated to date suggests that excess weight increases inflammation and oxidative stress, which have been associated with poorer executive function and lower self-regulation due to the indirect effect of the obesity-induced activation of immune responses [7]. In addition, evidence has shown that overweight and obesity may also induce insulin resistance, promoting neurodegeneration in the brain and cognitive decline [8]. 

The recent literature on cognitive function and brain dysfunctions in overweight and obesity reports that executive function may be particularly affected by excess weight [9,10]. A recent meta-analysis of 72 studies and 4904 participants with overweight and obesity found that a higher BMI was associated with an increased range of impairments in executive function, including deficits in inhibition, working memory, decision making, planning, and cognitive flexibility [10]. Similar findings have been reported in a previous systematic review [11], showing that obese individuals display poorer executive function in tasks related to planning, problem solving, or decision making than healthy individuals. Despite these findings, research has demonstrated that deficits in executive function (EF) due to excess weight are potentially modified by diet and physical exercise. For example, a meta-analysis of 20 randomized controlled trials (RCTs) and longitudinal studies evaluating the influence of voluntary weight loss on cognitive function in adults with overweight and obesity confirmed these findings, suggesting that weight loss may induce improvements in cognitive domains related to attention, memory, and language processes [12]. 

Since impairments in cognitive function are associated with nutritional habits, it is important to examine how a high BMI affects cognitive function and the specific deficits displayed by participants with overweight and obesity. Understanding deficits in executive function may help to inform clinicians and researchers about rehabilitation efforts as well as facilitating the development of nutritional interventions focused on improving nutritional habits as well as physical and mental health conditions in adults with overweight and obesity. 

The present study aimed to examine the association between body mass index and executive function in adult individuals with overweight and obesity. In particular, we examined the potential associations of markers of adiposity (BMI, body fat, and visceral fat) with five domains of EF, namely, cognitive flexibility, inhibition, monitoring, planning, and working memory. We hypothesized that higher body fat and visceral fat would be associated with poorer executive function and that obese individuals would show more impairments in all the domains of EF. 

## 2. Materials and Methods

### 2.1. Study Participants and Procedure

The participants included 87 male and female Spanish volunteers with overweight and obesity recruited by advertisements on the website of the Tech4Diet project: 4D modelling and visualization of the human body (http://tech4d.dtic.ua.es/ (accessed on 22 June 2021)). The participants ranged in age from 22 to 63 years (M = 47.14 years; SD = 9.22 years). The inclusion criteria were (i) having a body mass index (BMI) greater than 25 kg/m^2^, (ii) being right-handed, (iii) being able to read and write fluently, and (iv) having Spanish as the mother tongue. The exclusion criteria were (i) currently being or having in the past year been on a dietary/nutritional treatment supervised by a nutritionist; (ii) the presence of endocrinometabolic disorders including problems of the thyroid, pituitary gland, or adrenal gland and metabolic syndrome; (iii) having a prior history of neurological illness (e.g., stroke or Parkinson’s disease); (iv) having a history of head injury (causing a loss of consciousness for more than 30 min); (v) having a history of severe psychopathology according to the DSM-IV-TR diagnostic criteria; and (vi) currently receiving psychiatric treatment. Initial participants were recruited from September to November 2020. From the 101 individuals approached, 14 (16.09%) were excluded due to meeting exclusion criteria: five (5.75%) had followed dietary treatments over the past year, six (6.9%) had histories of endocrinometabolic disorders, two (2.3%) were taking psychopharmacological medications due to mental health disturbances, and one (1.15%) had a history of head injury. The final sample included 87 male and female participants with overweight and obesity. The measurements were conducted on one testing day. Additionally, all the participants completed a neuropsychological battery of executive function tests. 

### 2.2. Ethical Considerations

The study was approved by the Ethics Committee of the Instituto de Investigación Sanitaria y Biomédica de Alicante (ISABIAL (Health and Biomedical Research Institute of Alicante)) (CEIm: 180380). The participants were informed about the study, the voluntary nature of their participation, and the fact that they could withdraw from the study with no consequences. Informed consent was obtained from all the subjects involved in the study.

### 2.3. Anthropometrics, Body Composition, and Clinical Parameters

The body weights (0.1 kg precision) and heights (0.1 cm precision) of the participants were measured with them wearing light clothing and no shoes. A digital weighing scale, TANITA MC-780MA P (TANITA Corporation, Arlington Heights, IL, USA), and a SECA^R^ portable stadiometer 213 (SECA, Hamburg, Germany) were used to carry out the measurements. 

The waist and hip circumferences were measured using a flexible measuring tape (measurement precision, 0.1 cm). All the measurements were performed twice, and the mean values were calculated for data analysis. 

The body mass index was calculated as weight/height squared (kg/m^2^), and the waist-to-hip ratio (WHR), as the ratio of the waist to hip circumference. We also examined the body fat percentage (%) and visceral fat area (cm^2^). BMI was interpreted according to the World Health Organization (WHO) classification. The BMI cut-off point for overweight was defined as ≥ 24 kg/m^2^, while obesity was defined as a BMI ≥ 30 kg/m^2^ [13]. We also examined capillary cholesterol, glucose, and TG concentrations with the Accutrend^®^ Plus using two drops of blood (15–40 μL) collected from different fingers, by using a lancing device (Accuchek^®^ Softclix^®^ Pro, Roche Diagnostics GmbH, Mannheim, Germany). 

### 2.4. Physical Activity 

Physical activity was determined by using the International Physical Activity Questionnaire Short Version (IPAQ-SF). The IPAQ-SF [14] comprises 7 items assessing the frequency and duration of physical activity across three ranges of intensity—vigorous physical activity (VPA = 8.0 metabolic equivalents (METs)), moderate physical activity (MPA = 4.0 METs), and low physical activity (LPA = 3.3 METs)—undertaken across a set of domains including leisure time, domestic and gardening (yard) activities, and work-related and transport-related activities during a typical week of one’s life. On the basis of the collected data on the frequency and duration of physical activity, we calculated the estimated energy expenditure (EE) and expressed it in METmin/week.

### 2.5. Cognitive Function

Executive function was examined by using the CogniFit^TM^ General Cognitive Assessment (CAB), which is a computer-assessed neuropsychological test battery used in research protocols for assessing cognitive function. The CogniFit (CAB) takes 30–40 min and measures a broad range of 15 cognitive areas including attention, perception, inhibition, monitoring, naming, planning, response time, recognition, shifting, spatial perception, updating, visual memory, working memory, visual scanning, and eye–hand coordination. Scores on the 15 cognitive abilities (http://www.cognifit.com (accessed on 22 June 2021)) are assigned using weights previously derived from a factor analyses performed on normative data and standardized into Z scores. The CogniFit neuropsychological battery has been widely used for clinical and research purposes [15,16,17]. It has been validated [18] against several standard neuropsychological tests, including the full Cambridge Neuropsychological Test Automated Battery (CANTAB), Raven´s Standard Progressive Matrices, the Wisconsin Card Sorting Test, the Continuous Performance Test, the STROOP test, and a variety of reading tests. The CogniFit scores range from 0 to 800 points, with higher scores indicating higher cognitive performance. In the present study, we used the executive function measures of the General Cognitive Assessment (CAB). In particular, we examined the following cognitive domains of executive function: cognitive flexibility (refers to the ability to adapt to a change or unexpected events), inhibition (ability to control impulsive and automatic responses and generate responses using attention and reasoning), monitoring (ability to complete a plan of action making possible to identify and correct any change from the original plan), planning (ability to think about future events and mentally anticipate the correct way to carry out a task or reach a specific goal), and working memory (ability to temporarily store and handle information in order to perform complex cognitive tasks). These set of abilities are mostly outlined by the prefrontal structures of the brain and can be trained and improved with practice and cognitive training. 

### 2.6. Statistical Analyses

Sociodemographic and clinical comparisons between the subjects with overweight and obesity were evaluated using chi-squared tests for the categorical variables, Student’s *t*-tests for the normally distributed continuous variables, and the Mann–Whitney *U* test for the skewed continuous variables. The association between the adiposity measures (BMI, body fat, and visceral fat) and executive function (CogniFit total score, cognitive flexibility, inhibition, planning, planning, and working memory) were analyzed using linear regression modeling. Assumptions of normality and multicollinearity were tested by the variance inflation factor; no violation of the assumptions was detected. All the statistical analyses were performed using SPSS IBM Corp. (released 2012; IBM SPSS Statistics for Windows, Version 21.0. Armonk, NY: IBM Corp), considering *p* < 0.05 to be significant. The descriptive values are expressed as the mean and standard deviation (M and SD, respectively). 

## 3. Results 

### 3.1. Sociodemographic Variables 

The sociodemographic data are presented in Table 1. There were no significant intergroup differences in sex, age, marital status, and current alcohol and tobacco consumption. The sample differed in terms of educational level, as shown in Table 1. 

### 3.2. Anthropometrics, Body Composition, and Clinical Parameters

Table 2 presents the anthropometrics, body composition, and clinical parameters of the participants. Obese group had higher weights (*p* ≤ 0.001), higher body fat (*p* = 0.02), higher visceral fat (*p* ≤ 0.001), and lower scores for physical activity (*p* = 0.03) than those with overweight. No significant differences were found in height and WHR. There were no significant intergroup differences in the clinical parameters of glucose, cholesterol, and triglycerides. 

### 3.3. Differences in Executive Function between Individuals with Overweight and Obesity

**Figure 1:**
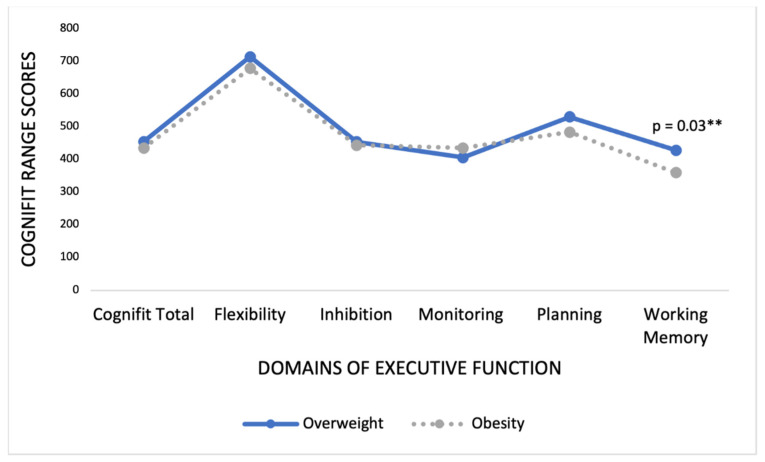
Differences in CogniFit scores between individuals with overweight and obesity. ** *p* < 0.001.

The differences in executive function between the participants with overweight and obesity are presented in Table 3 and Figure 1. There were no significant intergroup differences in the cognitive total score, flexibility, inhibition, monitoring, and planning. However, the individuals with overweight demonstrated better working memory than those with obesity *(t* = 2.08; *p* = 0.03). 

### 3.4. Anthropometrics, Body Composition, and Clinical Parameters

The relationships between executive function and the markers of adiposity (WHI, body fat, and visceral fat) are presented in Table 4. A lower body fat adiposity in the overweight individuals was significantly associated with two cognitive domains of executive function after adjusted by educational level and physical activity. In particular, lower body fat was associated with higher levels of inhibition (β = −0.35; 95% CI = 38.43, −1.96; *p* = 0.05) and monitoring (β = −0.44; 95% CI = −35.28, -2.80; *p* = 0.02). In the obese subgroup, lower body fat was associated with better inhibition (β = −0.32; 95% CI = −2.27, −0.14; *p* = 0.02) and working memory (β = 0.04; 95% CI = −0.68, 0.48; *p* = 0.04). 

## 4. Discussion

This study examined the potential associations of markers of adiposity (BMI, body fat, and visceral fat) with five cognitive domains of executive function. In particular, we examined the neuropsychological performance in a task related to brain executive function (flexibility, inhibition, monitoring, planning, and working memory) in adult with overweight and obesity. Participants with overweight and obesity showed similar cognitive function according to all the cognitive measures of executive function, with the exception of working memory, for which participants with obesity showed worse scores than those overweight. Working memory, which has been defined as the ability to keep information in the mind [19], is thought to play an important role in self-regulation and the emotional regulation of eating behavior [20,21]. Previous studies have reported that lower working memory is associated with a loss of control in eating and the choice of highly calorie-dense foods, particularly with higher snack food and fat intakes [22,23]. The association between obesity and lower working memory found in our study is consistent with previous studies that have suggested that working memory is frequently affected by a higher BMI [24]. Additionally, it has been reported that working memory in obesity is affected by inflammatory activity caused by the activation of immune system, which has been associated with impairments in neural processes that regulate the prefrontal cortex and are implicated in multiple processes of executive function [10,25,26]. Evidence from patients with dementia and laboratory studies in rodents have also demonstrated that adiposity and inflammation may alter brain structure, especially in obese patients, leading to a loss of synapses and deficits in the hippocampal region, which is directly involved in all memory processes [27]. Moreover, adiposity has also been associated with worse performance in young and older adults with normal weights in task switching, which requires working memory [28]. We also observed a linear relationship between lower body fat and better ability for inhibition in obese participants. Previous studies have reported that a lack of inhibition control results in impulsive behavior, overeating, and unhealthy behaviors [29], while increasing levels of inhibition may help patients to lose weight and maintain healthy behaviors [30]. Lower inhibitory control has been associated with resistance to change and an inability to modify food behaviors [31]. In our study, participants were voluntarily recruited from the community with the purpose of conducting an intervention program for weight loss on the basis of the beneficial effect of the Mediterranean diet. Therefore, these individuals might have had greater motivation for weight loss and less resistance to change than the general community.

The results of the present study suggest that lower body fat was significantly associated with cognitive function, after controlling for educational levels and daily physical activity. However, neither the waist–hip index nor visceral fat were significant predictors of executive function. Interestingly, lower body fat in particpants with overweight was positively associated with better function in executive components of inhibition and monitoring, more so than in obese participants. Evidence from independent studies shows that lower adiposity is associated with changes in cognition. Some executive components such as inhibition, monitoring, and planning are of critical importance, as they provide the self-regulation needed to execute goal-directed behavior and initiate a plan [32]. Research has demonstrated that executive function is inversely associated with body mass index, even in healthy individuals [33]. Therefore, it is possible that paticipants with reduced BMIs display greater abilities for self-control, self-monitoring, and maintaining energy balance compared to people with elevated body fat [34]. In the present study, body fat was a stronger predictor than visceral fat of cognitive function in both overweight and obese individuals. These results might indicate that, while visceral fat or abdominal obesity increase the adipose tissue surrounding the intra-abdominal organs [35], body fat might play a more direct role in other body organs such as the brain. Evidence from previous studies suggests that the lower body fat in adults with overweight, compared to those with obesity, might lead to a lower grade of inflammation in the brain and better brain connectivity [35,36]. Future studies should test this hypothesis. In addition, these results might also suggest the importance of examining the independent roles of body fat, visceral fat, and the waist–hip ratio rather than only BMI in the cognitive function of patients with overweight and obesity, given that the accumulation of fat in different regions of the body including the brain might suggest different profiles of cognitive impairment.

There are several limitations of the current study that suggest areas for future research. First, the study was cross-sectional, precluding the establishment of causal inferences. Second, we used a small sample size from a single city in Spain, and therefore researchers and interventionists must practice caution when generalizing the findings. Third, the participants in our study were voluntarily recruited from the community with the purpose of conducting an intervention program for weight loss based on the beneficial effects of the Mediterranean diet. Therefore, these individuals might have been more highly motivated to lose weight and less resistant to change than the general community. Finally, as suggested in previous studies, elevated adiposity may lead to impairments in cognitive function, but it is also possible that poor cognitive function may lead to an elevated BMI, given that a lack of inhibition in eating results in impulsive behavior and overeating [28]. Despite these limitations, this study provides evidence of the importance of adiposity in health and cognitive function.

## 5. Conclusions

The results provide evidence of the influence of markers of adiposity for cognitive function overweight and obesity. In particular, our findings suggest the importance of examining the independent roles of body fat, visceral fat, and the waist-hip ratio rather than only BMI in the cognitive function of participants with overweight and obesity given that the accumulation of fat in different regions of body might suggest different profiles of cognitive impairment. 

## Figures and Tables

**Table 1:** Sociodemographic characteristics of participants (*n* = 87).

	Overweight (*n* = 34, SD)	Obese (*n* = 53, SD)	*p*
Sex			0.82
Female	21 (38.2)	34 (61.8)	
Male	13 (40.6)	19 (59.4)	
Age	46.12 ± 10.23	47.43 ± 8.37	0.10
Marital status			0.59
Single	6 (17.64)	5 (9.43)	
Married	24 (70.59)	41 (77.36)	
Divorced	4 (11.77)	6 (11.32)	
Other	0 (0.0)	1 (1.89)	
Educational level			0.01 *
Primary studies	1 (2.94)	6 (11.32)	
Secondary studies	7 (20.59)	24 (45.28)	
University studies	26 (76.47)	23 (43.40)	
Alcohol consumption			0.06
Current use	23 (70.59)	31 (64.15)	
No	10 (29.41)	19 (35.85)	
Tobacco consumption			0.28
Current use	6 (17.65)	9 (16.98)	
No	28 (82.35)	44 (83.02)	

* *p* < 0.01.

**Table 2:** Anthropometrics, body composition, and clinical parameters of participants (*n* = 87).

	Overweight (*n* = 34)	Obesity (*n* = 53)	Total (*n* = 87)	*p*
Weight	76.95 ± 10.00	99.28 ± 14.98	90.72 ± 17.15	<0.00 **
Height (m)	1.65 ± 0.09	1.66 ± 0.08	1.66 ± 0.09	0.60
WHR ^1^	0.87 ± 0.08	0.91 ± 0.11	32.83 ± 5.52	0.10
Body fat	30.72 ± 6.59	58.32 ± 83.16	48.10 ± 67.22	0.02 *
Visceral fat	8.93 ± 4.30	11.46 ± 4.80	11.78 ± 5.10	<0.00 **
Muscle mass	66.14 ± 6.37	68.57 ± 68.77	67.67 ± 54.60	0.84
Glucose	83.69 ± 21.84	80.33 ± 14.32	81.55 ± 17.37	0.41
Cholesterol	208.86 ± 40.00	201.28 ± 32.17	204.06 ± 35.18	0.36
Triglycerides	266.39 ± 183.21	251.91 ± 151.58	255.26 ± 157.27	0.80
IPAQ ^2^ (MET/minute/week)	2618.36	1216.82	1729.58 ± 2821.72	0.03 *

^1^ WHR: waist–hip ratio; ^2^ IPAQ: physical activity questionnaire; * *p* < 0.01; ** *p* < 0.001.

**Table 3:** Differences in executive function between overweight and obesity (*n* = 87).

	Overweight (*n* = 34)	Obesity (*n* = 53)	Total (*n* = 87)	*t*	*p*
CogniFit total	455.71 ± 114.65	436.62 ± 114.13	444.08 ± 114.05	7.70	0.45
Flexibility	715.09 ± 138.26	679.98 ± 154.05	693.70 ± 148.26	1.07	0.28
Inhibition	455.12 ± 341.42	444.81 ± 324.79	449.93 ± 329.42	0.01	0.99
Monitoring	407.06 ± 287.34	436.57 ± 267.75	425.03 ± 274.30	−0.48	0.63
Planning	531.18 ± 202.14	485.08 ± 218.75	503.09 ± 212.43	0.98	0.33
Working memory	429.38 ± 129.44	360.66 ± 161.25	387.52 ± 152.60	2.08	0.03 **

** *p* < 0.01.

**Table 4:** Associations of executive function and markers of adiposity.

	WHR		Body Fat (%)		Visceral fat	
	Standardized β (95% CI)	*p*	Standardized β (95% CI)	*p*	Standardized β (95% CI)	*p*
CogniFit total score						
Overweight	0.33 (−83.70, 98.10)	0.86	−3.81 (−12.69, −1.10)	0.07	−0.13 (−13.61, 7.17)	0.51
Obesity	−0.06 (−83.65, 54.17)	0.66	−0.12 (−0.55, 0.23)	0.42	0.01 (−7.41, 7.10)	0.96
Cognitive flexibility						
Overweight	0.07 (−101.18, 144.55)	0.72	−0.09 (−11.21, 7.21)	0.65	−0.18 (−19.12, 7.51)	0.37
Obesity	−0.21 (−143.47, 126.35)	0.17	−0.09 (−0.06, 0.33)	0.51	0.08 (−6.46, 11.42)	0.58
Inhibition						
Overweight	−0.12 (−54.54, −83.93)	0.52	−0.35 (−3.43, −1.96)	0.05	−0.22 (−46.91, 11.45)	0.22
Obesity	0.26 (−14.55, −35.09)	0.07	−0.32 (−2.27, −0.14)	0.02	0.15 (−8.51, 30.40)	0.26
Monitoring						
Overweight	0.01 (−207.61, 225.42)	0.93	−0.44 (−35.28, −2.80)	0.02	−0.28 (−41.97, 4.96)	0.12
Obesity	0.17 (−62.75, 156.53)	0.23	−0.22 (−1.68, 0.15)	0.10	0.17 (−6.71, 26.40)	0.23
Planning						
Overweight	0.10 (−129.92, 212.05)	0.62	−0.37 (−24.29, 1.36)	0.07	0.10 (−13.72, 23.34)	0.59
Obesity	0.12 (−225.97, 42.25)	0.17	−0.01 (−0.07, 0.77)	0.99	0.17 (13.34, 14.89)	0.91
Working memory						
Overweight	0.02 (−102.31, 114.57)	0.91	−0.60 (−0.68, 0.48)	0.79	−0.17 (−16.69, 6.81)	0.39
Obesity	−0.15 (−126.18, 66.48)	0.53	−0.09 (−1.88, 0.38)	0.04	−0.15 (−15.31, 4.92)	0.31

Adjusted by educational level and physical activity.
